# Aberration retrieval by incorporating customized priors for estimating Zernike coefficients

**DOI:** 10.1038/s41598-020-68012-3

**Published:** 2020-07-07

**Authors:** Bin Wang, Xiaofei Wang, Qichang An

**Affiliations:** 1 Key Laboratory for Applied Statistics of MOE, School of Mathematics and Statistics, Northeast Normal University, Changchun, 130024 China; 2 Changchun Institute of Optics, Fine Mechanics and Physics, Chinese Academy of Sciences, Changchun, 130033 China; 3 Key Laboratory of Space Object and Debris Observation, PMO, CAS, Nanjing, China

**Keywords:** Optics and photonics, Mathematics and computing

## Abstract

Zernike expansion is an important tool for aberration retrieval in the optical field. The Zernike coefficients in the expansion can be solved in a linear system from those focal region intensity images, which can be modeled by the extended Nijboer–Zernike approach. Here we point out that those coefficients usually follow from different prior distributions, and especially, their variances could be dramatically diverse. To incorporate the prior information, we further introduce customized penalties to those Zernike coefficients and adopt a global adaptive generative adjustment algorithm for estimating coefficients. Based on both simulated and real data, numerical experiments show that our method outperforms other conventional methods, and provides an estimate of Zernike coefficients with a low mean square error.

## Introduction

The aberration retrieval (AR) from the intensity point-spread function in the focal region is widely used in the optical field. It usually adopts the Zernike expansion to represent the aberration linearly. The phase retrieval^1,2^, phase diversity^3,4^ and curvature sensing^5,6^ are three classical methods for the AR. They solve inverse problems based on the optical mechanism and statistical parameter estimation. The work^7^ considered an extended Nijboer–Zernike (ENZ) diffraction from an analytic description of the focal field and realizable solutions for the aberration coefficients^7–9^. A further work^10^ suggested an ENZ AR method for identifying the imperfection of lens from the intensity point spread function (PSF) of the optical system. This ENZ AR method can be further applied to the high-resolution optical lithography^11^. The general pupil function can be represented by a linear function of Zernike coefficients in the Zernike expansion. Through further diffraction integrals, the light field on the focal plane also has an expansion:

1 $$\begin{aligned} U(r,\varphi ;f) = 2\sum _{n,m} {\mathbf {i}}^m \beta _n^m V_n^m(r,f)\exp ({\mathbf {i}}m\varphi ), \end{aligned}$$

in which the pair $(r,\varphi )$ are polar coordinates on the image plane, the parameter *f* is the camera distance from the focus plane, $\{V_n^m(r,f)\}_{n,m}$ are contants relying on the optical system given *r* and *f*, and coefficients $\{\beta _n^m\}$ are extended Nijboer–Zernike coefficients.

**Figure 1:**
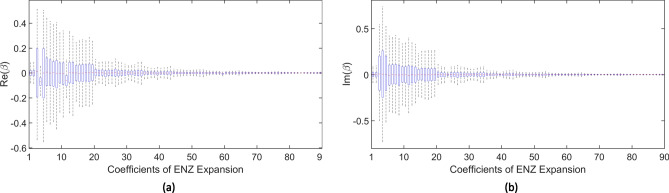
The box plot for 90 Zernike coefficients. (**a**) Real part of coefficients. (**b**) Imaginary part of coefficient.

**Figure 2:**
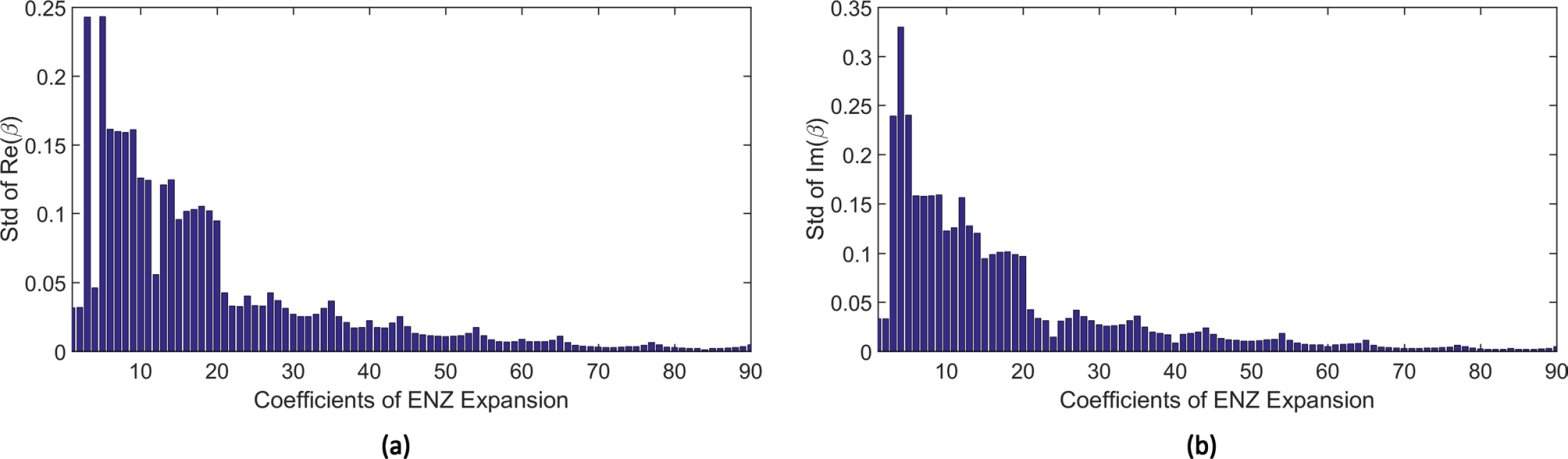
The standard deviation for 90 Zernike coefficients. (**a**) Real part of coefficients. (**b**) Imaginary part of coefficient.

In theory, the expansion considers infinite terms, but in practice, only the first Q terms are retained for the aberration retrieval. For example, Q $=$ 91 was chosen to estimate those Zernike coefficients in the work^12^. Moreover, it illustrates that those coefficients could follow some prior distribution and introduce the penalty mechanism into the aberration retrieval in the optical field. Here we further study the prior distribution of the Zernike coefficients by decomposing the simulated pupil function. As discussed in the work^12^, atmospheric wavefronts can be simulated using the method^13^ to generate 1000 random phases. Furthermore, 1000 generalized pupil functions can be generated with those phases at constant amplitude. We consider the first 91 terms in the Zernike expansion of the generalized pupil function. Since the first term has a real coefficient, there are 181 real coefficients by separating the real and imaginary parts. We set the first coefficient to be one, and compute the other 180 coefficients. By decomposing 1000 simulated pupil functions, we obtain 1000 observations for each coefficient. The box plot on Zernike coefficients is shown in Fig. 1. This box plot illustrates that those coefficients follow from different prior distributions. Moreover, their standard deviation could be dramatically diverse as shown in Fig. 2. Inspired by these findings, we assume that Zernike coefficient $\beta _{n}^{m}$ follows a customized prior distribution, which is Gaussian with mean zero and variance $\sigma _{n,m}^2$. The prior mentioned in our paper means a knowledge that the prior distributions for various model parameters could have diverse variances. Since we consider the Gaussian prior with zero mean, the prior distribution is determined only by its variance. The “customized priors” mean the customized variances for model parameters.

For incorporating the prior information, we further introduce customized penalties for those Zernike coefficients and adopt a global adaptive generative adjustment algorithm for estimating coefficients. The experiment results on the simulated and real data illustrate that our method, utilizing customized prior variances, provides an estimate with a lower mean squared error compared to other ENZ aberration retrieval methods.

## Methods

### Extended Nijboer–Zernike diffraction

In this study, we assume that the optical system is monochromatic and its aperture is circular and unobstructed. The generalized pupil function is usually expressed as^14^

2 $$\begin{aligned} Pupil(\rho ,\theta )=A(\rho ,\theta )\exp (\mathbf{i }\phi (\rho ,\theta )) \end{aligned}$$

where $A(\rho ,\theta )$ and $\phi (\rho ,\theta )$ are the amplitude and phase of the pupil plane, respectively. $\mathbf{i }=\sqrt{-1}$ and $(\rho ,\theta )$ are polar coordinates, $\rho \in [0,1]$, $\theta \in [0,2\pi ]$. The phase can be linearly expressed by Frits Zernike expansion^14^

3 $$\begin{aligned} \phi (\rho ,\theta )=\sum \limits _{k=0}^{K-1}\alpha _k Z_k(\rho ,\theta ) \end{aligned}$$

where $\{Z_k(\rho ,\theta )\}_{k=0}^{K-1}$ are Frits Zernike basis functions, and $\{\alpha \}_{k=0}^{K-1}$ are the coefficients of the Zernike basis. From the work^15^, the generalized pupil function can be decomposed by using Zernike coefficients $\{\beta _n^m\}$ as

4 $$\begin{aligned} Pupil(\rho , \theta ) = \sum \limits _{n,m} \beta _{n}^m R_n^{|m|}(\rho )\exp ({\mathbf {i}}m\theta ), \end{aligned}$$

where *m* and *n* are integers such that $n \ge 0$ and $n-|m|$ is even. And $R_n^{|m|}(\rho )$ is the radial polynomial. The first Zernike coefficient $\beta _0^0$ is real, and the others $\{\beta _n^m\}$ are complex for $m \ne 0, n \ne 0$. In the simulation, we generated random phases $\{\alpha \}_{k=0}^{K-1}$ using the method^13^. Thus we also obtained the phase function $\phi (\rho ,\theta )$ by (3). Furthermore, we computed the generalized pupil function by (2) at constant amplitude. Finally, we consider the first 91 terms in the Zernike expansion (4) of the generalized pupil function.We denote all the Zernike coefficients by a coefficient vector $\varvec{\beta }$. Thus the pupil function is a linear function on the vector $\varvec{\beta }$, which is to be estimated as aberration retrieval.

From works^11,15^, the radial polynomials in (4) can be expressed as

5 $$\begin{aligned} R_n^{|m|}(\rho ) = \sum _{s=0}^{\frac{n-|m|}{2}} (-1)^s \frac{(n - s)!}{s!(\frac{n+|m|}{2}-s)!(\frac{n-|m|}{2}-s)!} \rho ^{n-2s}. \end{aligned}$$

Hence $R_n^{|m|}(\rho )$ can be constructed before aberration retrieval for a specific choice of *m*, *n*, and $\rho$.

In the work^8^, the light field on the focal plane is expressed as

$$\begin{aligned} U(r,\varphi ;f) = 2\sum _{n,m} {\mathbf {i}}^m \beta _n^m V_n^m(r,f)\exp ({\mathbf {i}}m\varphi ), \end{aligned}$$

where the parameter *f* is the camera distance from the focus plane, and $(r,\varphi )$ are polar coordinates on the image plane. From the work^7^, the Bessel series has the form

6 $$\begin{aligned} V_n^m(r,f)=\exp ({\mathbf {i}}f)\sum _{l=1}^{\infty }(-2{\mathbf {i}}f)^{l-1}\sum _{j=0}^{p}v_{lj}\frac{J_{|m|+l+2j}(2\pi r)}{l(2\pi r)^l}, \end{aligned}$$

where $J_m$ is a Bessel function of the first kind with order *m*, and

7 $$\begin{aligned} v_{lj} = (-1)^{\frac{n-m}{2}}(|m|+l+2j)\left( {\begin{array}{c}|m|+j+l-1\\ l-1\end{array}}\right) \left( {\begin{array}{c}j+l-1\\ l-1\end{array}}\right) \left( {\begin{array}{c}l-1\\ p-j\end{array}}\right) \bigg /\left( {\begin{array}{c}q+l+j\\ l\end{array}}\right) , \end{aligned}$$

where $$q=\frac{n+|m|}{2}$$, $$p=\frac{n-|m|}{2}$$, and $$l=1,2,\ldots ; j=0,\ldots ,p$$.

### Aberration retrieval model using the ENZ approach

Using the formula (1), the PSF intensity in the focal region can be expressed as

8 $$\begin{aligned} \begin{aligned} I(r,\varphi ; f)&= |U(r,\varphi ;f)|^2 \\&=4|V_0^0 (r,f)|^2(\beta _0^0)^2 + 8\beta _0^0 \sum _{n,m} {'Re}[\beta _n^m {\mathbf {i}}^m V_n^m (r,f)V_0^{0*}(r,f) \exp ({\mathbf {i}}m \varphi )] + C(r,\varphi ;f) \\&=4|V_0^0 (r,f)|^2(\beta _0^0)^2 + 8\beta _0^0 \sum _{n,m} {'Re}(\beta _n^m)Re[{\mathbf {i}}^mV_n^m (r,f)V_0^{0*}(r,f)]\cos (m\varphi ) \\&\quad -\,8\beta _0^0 \sum _{n,m} {'Re}(\beta _n^m) Im[{\mathbf {i}}^m V_n^m (r,f)V_0^{0*}(r,f)] \sin (m\varphi )\\&\quad -\,8\beta _0^0 \sum _{n,m} {'Im}(\beta _n^m) Re[{\mathbf {i}}^m V_n^m (r,f)V_0^{0*}(r,f)] \sin (m\varphi ) \\&\quad -\,8\beta _0^0 \sum _{n,m} {'Im}(\beta _n^m) Im[{\mathbf {i}}^m V_n^m (r,f)V_0^{0*}(r,f)] \cos (m\varphi ) + C(r,\varphi ;f), \end{aligned} \end{aligned}$$

where

9 $$\begin{aligned} C(r,\varphi ;f) = 4 \sum \limits _{n_1,m_1;n_2,m_2} {''Re}\{\beta _{n_1}^{m_1} \beta _{n_2}^{m_2 *} {\mathbf {i}}^{m_1-m_2}V_{n_1}^{m_1}V_{n_2}^{m_2 *}\exp [{\mathbf {i}}\varphi (m_1-m_2)]\} \end{aligned}$$

is the sum of the remaining second order cross terms. In the summand, symbol $'$ means the omission of $n=0$ terms in the summand, and symbol $''$ means the omission of $n_1=m_1=0$ or $n_2=m_2=0$ terms. *Re*() and *Im*() denote the real and imaginary parts of a complex number. Symbol $^*$ denote the complex conjugate.

**Figure 3:**
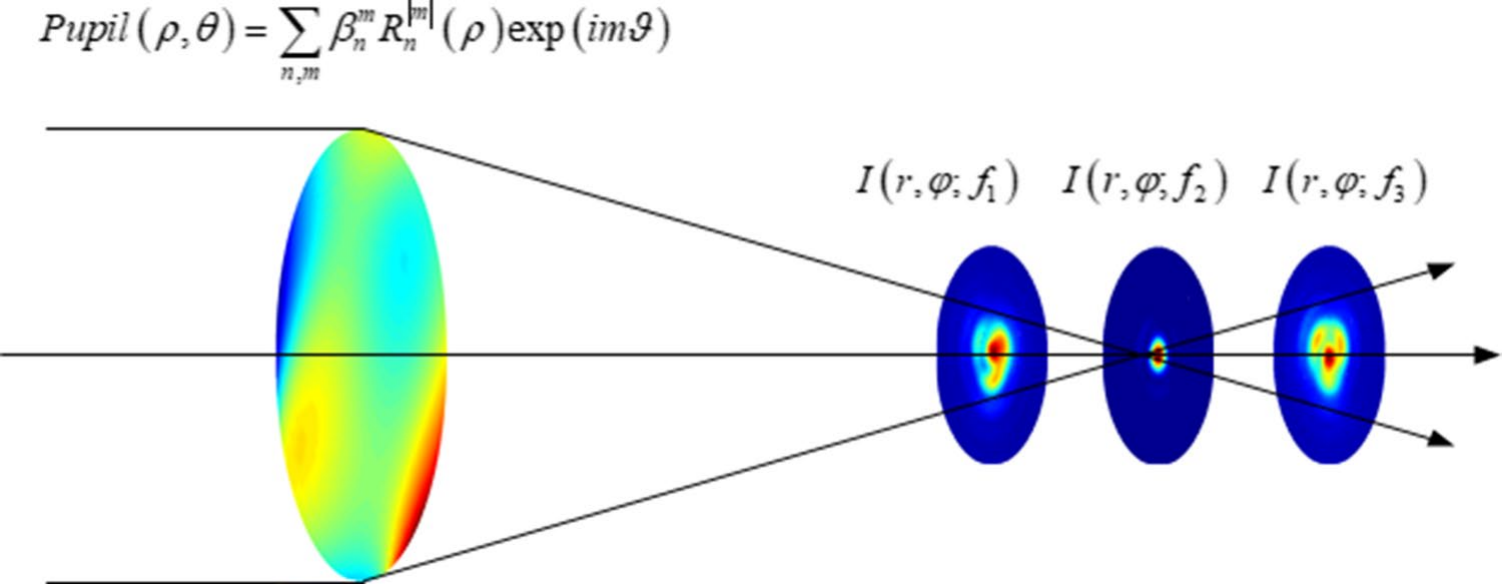
The generalized pupil and the through focus light field can be linearly expanded as a combination of coefficients $\{\beta _n^m\}$. The measured through focus PSFs are inputs for estimating coefficients $\{\beta _n^m\}$.

The PSF intensity with an additive detector readout noise $\varepsilon$ forms an aberration retrieval model:

10 $$\begin{aligned} I_{b}(r,\varphi ;f)=I(r,\varphi ;f)+\varepsilon (r,\varphi ;f), \end{aligned}$$

where the noise $\varepsilon (r,\varphi ;f) \sim N(0,\sigma ^2)$. The intensity $I_{b}(r,\varphi ;f)$ can be collected to estimate coefficients $\{\beta _n^m\}$ using (8), as shown in Fig. 3.

This aberration retrieval process, previously proposed in works^10,16–19^, has four main steps:

1. Input the collected PSFs $I_b$. Set the maximum iteration step *K*, $I^{(0)}=I_b$, $C^{(0)}=0$ and $k=0$.
2. Assumes that $I^{(k)}$ can be described as linear combinations of the entrance pupil aberrations with coefficients $\{\beta _n^m\}$. This is equivalent to omitting the cross terms of (8). And then compute $\{\beta _n^m\}^{(k)}$.
3. Calculate $C^{(k+1)}$ using (9) by $\{\beta _n^m\}^{(k)}$.
4. Set $I^{(k+1)} = I_b - C^{(k+1)}$, $k=k+1$. If $k > K$, exit. Otherwise, go to step (2).

Notice that due to phase wrapping effects occurring in the reconstructed pupil distribution, this retrieval process may fail in case that the aberration magnitude is beyond some large range. So we only considered a small wavefront error $(pv<2\pi )$ in our experiment. From works^10,12,16,17^, we know that the intensity $I^{(k)}$ in Step (2) can be linearly transformed into a vector ${\mathbf {L}}^{(k)}$. We further get a classical linear model on the coefficient vector $\varvec{\beta }$:

11 $$\begin{aligned} {\mathbf {L}}^{(k)}={\mathbf {M}}\varvec{\beta } + \varvec{\delta }, \end{aligned}$$

where $\varvec{\delta }$ is a Gaussian random noise with zero mean. The specific forms of ${\mathbf {M}}$, $\varvec{\beta }$, $\varvec{\delta }$, ${\mathbf {L}}^{(k)}$ can be referred to the work^12^.

Usually, the least square estimate:

12 $$\begin{aligned} \hat{\varvec{\beta }}^{(k)}=\arg \min _{\varvec{\beta }} \Vert {\mathbf {M}}\varvec{\beta }-{\mathbf {L}}^{(k)}\Vert _2^2 = \left( {\mathbf {M}}^T{\mathbf {M}} \right) ^{-1}{\mathbf {M}}^T{\mathbf {L}}^{(k)}. \end{aligned}$$

is used to deal with this retrieval process^10,16,17^. Recently, considering the potential prior information, the work^12^ further introduces the penalty mechanism

13 $$\begin{aligned} \hat{\varvec{\beta }}^{(k)}=\arg \min _{\varvec{\beta }} \Vert {\mathbf {M}}\varvec{\beta }-{\mathbf {L}}^{(k)}\Vert _2^2 + \mu \Vert \varvec{\beta }\Vert _1 \end{aligned}$$

into the retrieval process, and propose a penalized ENZ AR algorithm.

### Global adaptive generative adjustment for estimating coefficients

As shown in the “Introduction” section, those Zernike coefficients follow from different prior distributions. Especially, their standard deviation could be dramatically diverse. So we assume that coefficient $\beta _j$ has a prior $N(0,\tau _j^2)$ and the standard deviation $\tau _j$ could be diverse. From the classical linear model, the observed response ${\mathbf {y}}$ is generated by a linear system ${\mathbf {X}}\varvec{\beta }+\varvec{\epsilon }$, where $\varvec{\beta }=(\beta _1,\ldots ,\beta _p)^T$ can be viewed as the true signal, the Gaussian noise $\varvec{\epsilon }\sim N({\mathbf {0}},\sigma ^2{\mathbf {I}})$ and ${\mathbf {I}}$ is an identity matrix.

Considering the posterior distribution of coefficients, we further obtain an objective function

14 $$\begin{aligned} \frac{1}{2}\frac{\Vert {\mathbf {y}}-{\mathbf {X}}\varvec{\beta }\Vert ^2}{\sigma ^2}-\frac{n}{2}\log (\sigma ^2)+\frac{1}{2}\sum _{j=1}^{p} b_j{\beta _j}^2 \end{aligned}$$

with multiple hyperparameters $\{b_j\}$. These hyperparameters can be viewed as the prior information of model parameters $\{\beta _j\}$. In (14), *n* is the sample size, *p* is the dimension of the vector $\varvec{\beta }$, and the hyperparameter $b_j$, $j=1,\ldots ,p,$ provides a customized shrinkage on the coefficient $\beta _j$.

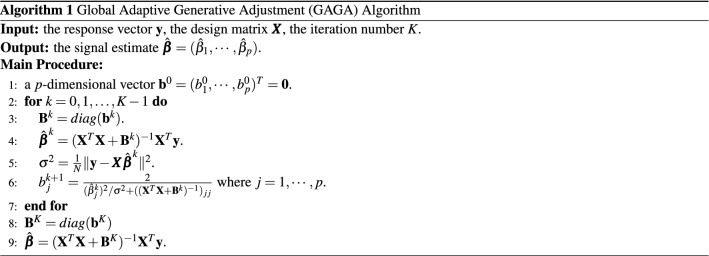



We adopt a global adaptive generative adjustment (GAGA) algorithm to recover a true signal $\varvec{\beta }$. In Algorithm 1, hyperparameters and the signal are alternatively updated by a data-driven method. The inputs of this algorithm are the response vector ${\mathbf {y}}$, the design matrix ${\mathbf {X}}$, the iteration number *K*. The output of this algorithm is the signal estimate $\hat{\varvec{\beta }}=\text {GAGA}({\mathbf {y}},{\mathbf {X}},K)$. The convergency analysis of Algorithm 1 and the large sample properties of its output is discussed in the work^20^.

The following Algorithm 2 combines the ENZ AR process and the GAGA algorithm, which utilizes the customized prior information and updates model parameters and hyperparameters alternatively.

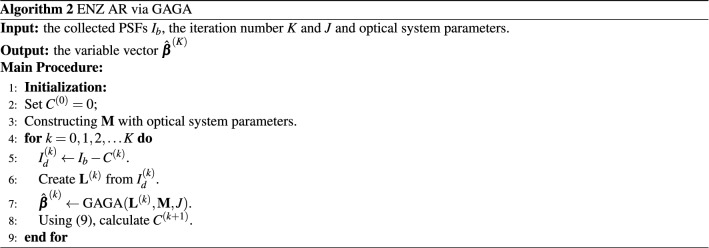



## Results

We suggest a method adopting a global adaptive generative adjustment (GAGA) algorithm for estimating the ENZ coefficients. We call this method the GAGA ENZ AR. In a previos work^12^, the least absolute shrinkage and selection operator (Lasso) algorithm can also be applied to the aberration retrieval. So we compared the GAGA ENZ to the Lasso ENZ AR and the conventional ENZ AR^17^ in the simulation with synthesized data. The characteristics of the optical system are shown in Table 1.

**Table 1:** Characteristics of the optical system.

Light source diameter (μm)	0.25
Numerical aperture	0.5
Wavelength (μm)	0.2
Polar angle sampling (°)	10
Polar radius sampling	$4pix/(\lambda F\#)$
Expected focus *f* (μm)	$-\,1/0/1$

The simulations were implemented in three steps:

1. We simulated three PSFs (images intra, in, and extra focus) from (8) with the first *Q* leading terms $\{\beta _n^m\}$, where $\beta _0^0 = 1$. In this paper, we set $Q=91$ in the AR process. We further added Gaussian white noise to PSFs and simulated four noise levels (40 dB, 35 dB, 30 dB, 25 dB) measured using Signal-Noise Ratio (SNR).
2. Using the simulated images with noise, we estimated $\{\beta _n^m\}$ by ENZ AR, Lasso ENZ AR and GAGA ENZ AR separately.
3. We divided the estimates $\{{\hat{\beta }}_n^m\}$ by ${\hat{\beta }}_0^0$ to ensure that the first component of $\{{\hat{\beta }}_n^m\}$ is 1, and then assessed the experimental results on the residual square error ${\Vert \hat{\varvec{\beta }}-\varvec{\beta }\Vert _2^2}$, where $\varvec{\beta }$ was the true parameter vector, and $\hat{\varvec{\beta }}$ was obtained by ENZ AR, Lasso ENZ AR and GAGA ENZ AR separately.

Different noise levels were chosen, and the above procedures were executed hundreds of time for each noise level. The empirical MSE of the true parameter $\varvec{\beta }$ were calculated by (15):

15 $$\begin{aligned} {MS\!E} = \frac{1}{N}\sum _{i=1}^{N}\Vert \hat{\varvec{\beta }}_i-\varvec{\beta }_i\Vert _2^2, \end{aligned}$$

where *i* means the *i*th test. *N* is the total number of test. The standard deviation of MSE can be further computed by:

16 $$\begin{aligned} {S\!T\!D} = \sqrt{\frac{1}{N-1}\sum _{i=1}^{N}\left( \Vert \hat{\varvec{\beta }}_i-\varvec{\beta }_i\Vert _2^2 - MS\!E\right) }. \end{aligned}$$

### Random aberration examples

**Figure 4:**
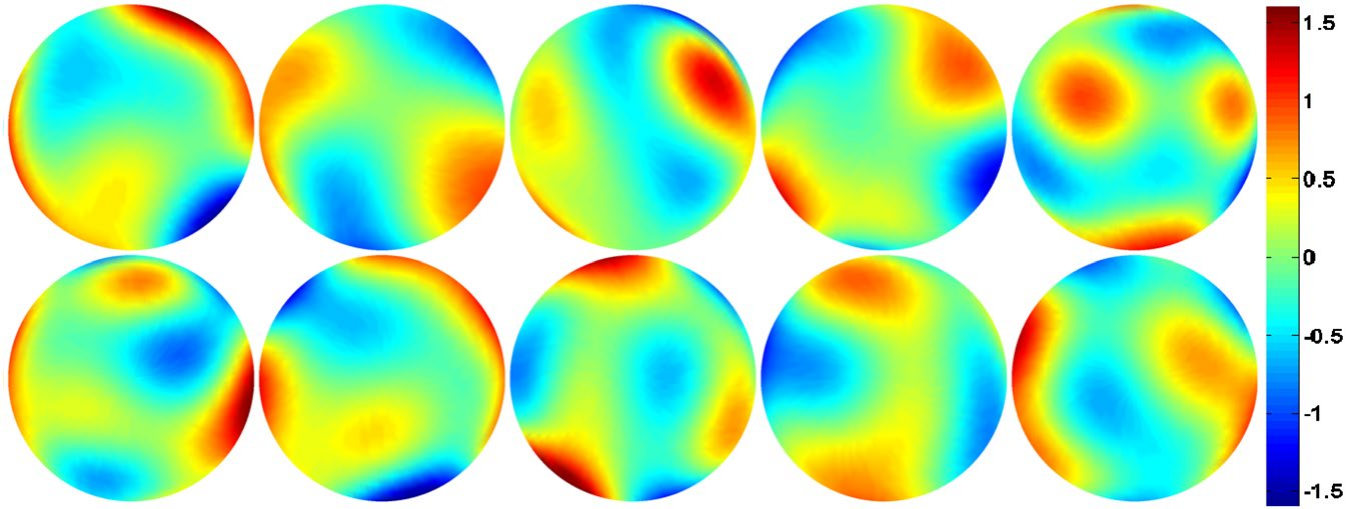
Some simulated random phases.

**Figure 5:**
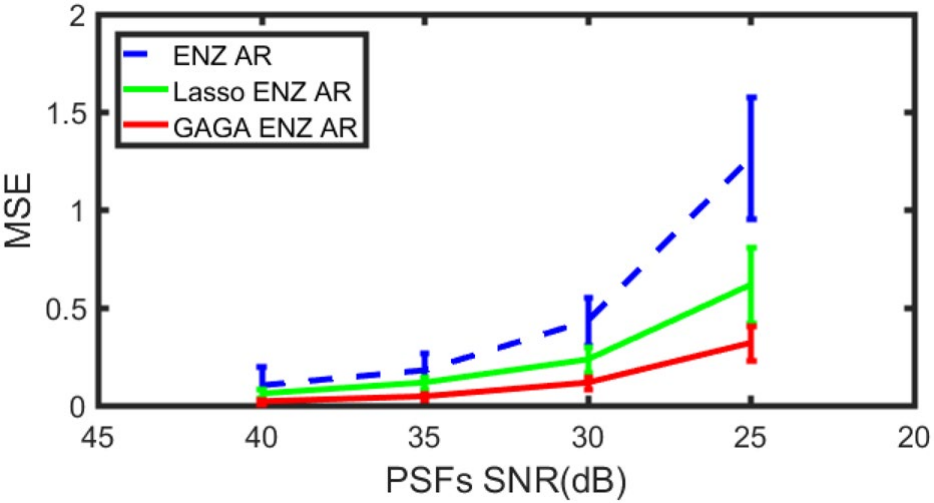
The error bar of three methods at each noise levels.

We used the method^13^ to generate 100 random phases $(pv<2\pi , rms=0.2\pi )$ by the first 21 terms of Frits Zernike expansion. Some of them are shown in Fig. 4. These phases at constant amplitude further generated 100 generalized pupils, which can be expressed by (4) for $Q=91$ in ENZ. Then we get 100 groups of $\{\beta _n^m\}$ as the true parameters in experiments. We compared the empirical MSE (15) for the output of ENZ AR, Lasso ENZ AR and GAGA ENZ AR. In Fig. 5 we show the error bar of the empirical MSE at each given SNR, where the length of the bar is two times the standard deviation STD (16). Our method GAGA ENZ AR produced superior parameter estimates at each noise level compared to ENZ AR and Lasso ENZ AR. Though our algorithm works well for a small wavefront error $(pv<2\pi )$, it may fail to handle the AR for strong wavefront aberrations in the atmospheric disturbances.

### Real data example

**Figure 6:**
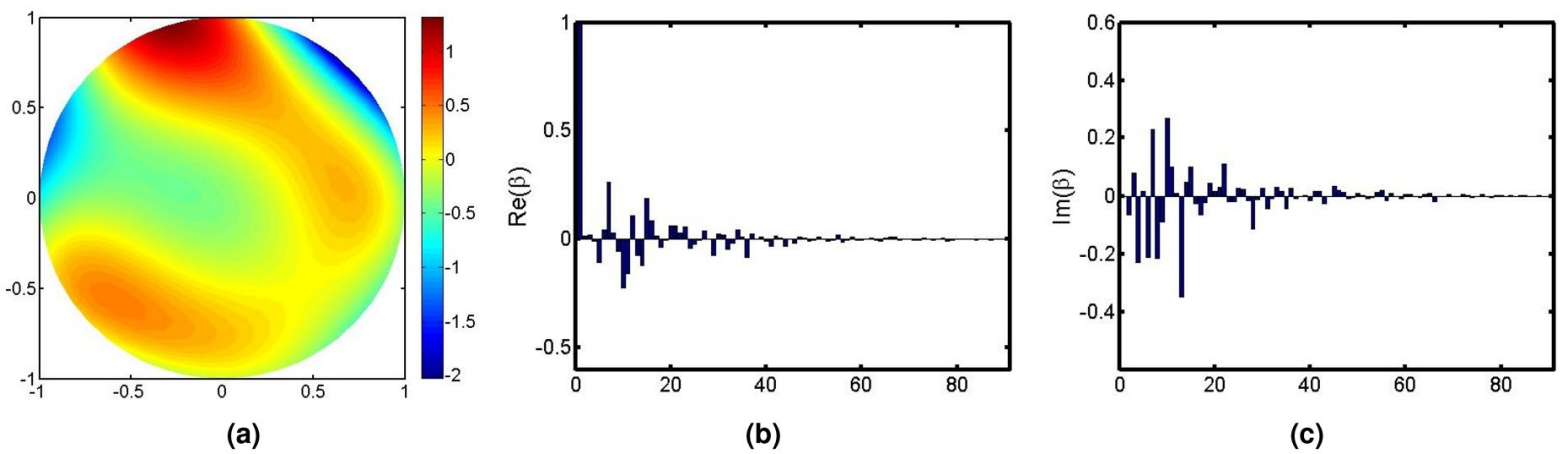
Phase of non-common path aberrations and NZ coefficients (representing the field with constant amplitude and the given phases). (**a**) Phases. (**b**) Real part of NZ coefficients. (**c**) Imaginary part of NZ coefficients.

The phase shown in Fig. 6a was observed from the measurement of non-common path aberrations from a 1.23 m adaptive optics telescope in Changchun China. More detailed descriptions on this optics telescope can be found in these works^21–23^. The field with constant amplitude and phase (Fig. 6a, $rms = 0.14\pi$) has NZ coefficients shown in Fig. 6b,c.

**Figure 7:**
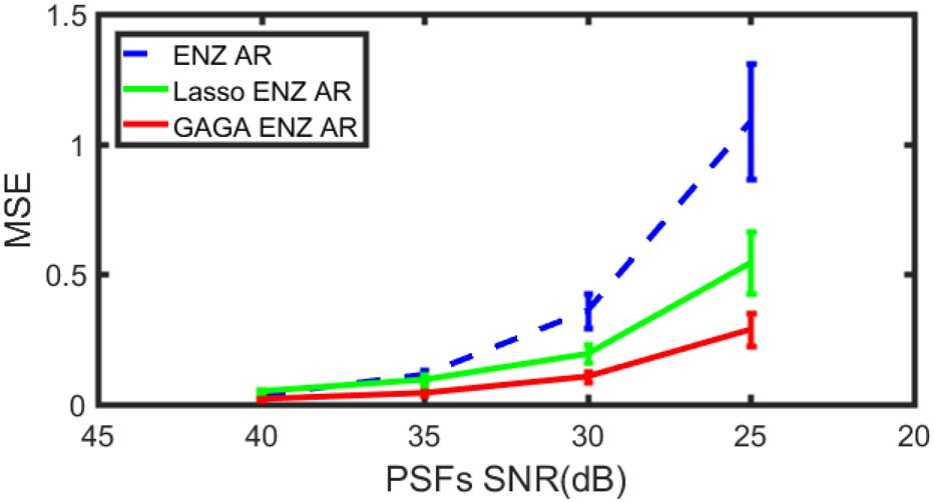
The error bar of three methods at each noise levels for the aberration shown in Fig. 6.

We repeated the simulation one hundred times and showed the error bar of the MSE in Fig. 7.

When the noise SNR is 40, the conventional ENZ AR performed marginally than LASSO ENZ AR. However, GAGA ENZ AR outperformed ENZ AR and LASSO ENZ AR at each noise level. Moreover, GAGA ENZ AR also kept the empirical MSE in a low value even at a high noise level.

## Conclusion

We find that to represent an optical field, Zernike coefficients have their customized priors. For incorporating the customized prior information, we adopt a global adaptive generative adjustment method for estimating coefficients in the ENZ aberration retrieval. In the simulated and real data experiments, our algorithm provides better performance on the MSE compared to ENZ AR and LASSO ENZ AR.
